# Inhibition of CXCR4 suppresses bone invasion in a murine model of oral squamous cell carcinoma

**DOI:** 10.1186/s12903-025-07544-4

**Published:** 2025-12-18

**Authors:** Letian Liu, Yusi Huang, Qi Zhang, Ke Zheng, Guoxin Yan

**Affiliations:** 1 Department of Stomatology, Wuxi No. 2 People’s Hospital, 68 Zhongshan Road, Wuxi, Jiansu 214125 P.R. China; 2 Department of Stomatology, Jiangyin People’s Hospital, Wuxi, Jiangsu 214499 P.R. China; 3 Department of Pathology, Wuxi No. 2 People’s Hospital, Wuxi, Jiangsu 214125 P.R. China

**Keywords:** C-X-C chemokine receptor type 4, Oral squamous cell carcinoma, Bone invasion, Epithelial-mesenchymal transition, Osteoclast activation

## Abstract

**Background:**

C-X-C chemokine receptor type 4 (CXCR4) plays a significant role in the tumor microenvironment of oral squamous cell carcinoma (OSCC), but its specific function in bone invasion remains poorly understood.

**Methods:**

Bioinformatic analysis of the GSE30784 dataset and Gene Ontology enrichment were performed. The role of CXCR4 was investigated using an in vivo model where poorly differentiated HSC-3 cells were inoculated into the heads of T cell-deficient female BALB/c nude mice (6 weeks old). Mice were treated systemically with the CXCR4 inhibitor AMD3100 (100 µl per dose, every other day for one week) or a PBS control (*n* = 3 per group). Bone invasion was defined as radiographically and histologically confirmed osteolysis of the bone. It was quantified by micro-computed tomography (micro-CT) analysis of parameters including Bone Mineral Density (BMD) and Bone Volume/Tissue Volume (BV/TV). Osteoclast activation and the expression of CXCR4 and epithelial-mesenchymal transition (EMT) markers were assessed by histochemical and immunohistochemical staining.

**Results:**

CXCR4 was upregulated in OSCC tissues and associated with biological processes critical for bone invasion, including cell motility and migration. In the murine model, AMD3100 treatment significantly suppressed mandibular bone destruction. Micro-CT analysis revealed a significant increase in BMD and BV/TV, and a decrease in Bone Surface/Bone Volume (BS/BV) in the treated group compared to controls (all *p* < 0.05). Histological evaluation showed that AMD3100 reduced bone resorption and decreased the number of TRAP-positive osteoclasts by approximately 80% (*p* < 0.01). Furthermore, immunohistochemical staining demonstrated that AMD3100 not only inhibited CXCR4 but also significantly downregulated the expression of the pro-invasive marker MMP9 and the EMT regulators Snail and Vimentin, with IHC scores reduced by 30–50% (*p* < 0.05).

**Conclusions:**

Our findings demonstrate that inhibition of CXCR4 suppresses bone invasion by OSCC through dual mechanisms: inhibiting osteoclast activation and impairing the EMT process in cancer cells. These results suggest that targeting CXCR4 represents a promising therapeutic strategy to prevent bone destruction in patients with OSCC.

## Introduction

The oral squamous cell carcinoma (OSCC) is the sixth most common cancer worldwide, the risk of which is greatly heightened by smoking, alcohol and betel nut chewing [1, 2]. Despite advances in its diagnosis and treatment, its 5-year survival remains low with poor prognosis [3]. Due to the oral epithelium being close to the mandible, oral cancer from the tongue, posterior area of molars and floor of mouth frequently invades the mandible, with the occurrence rate being reported to be 42, 48 and 62%, respectively [4]. The pattern of OSCC bone invasion can be divided into the following three patterns: Erosive type, expensive type and infiltrative type. In the erosive type, there is typically a stroma between bone and cancer. In the expansive type, the cancer will typically touch the bone directly, with a sharp interface. By contrast, in the infiltrative type, the cancer will touch the bone with an irregular interface [5]. The erosive type requires edge surgery control, whilst the infiltrative type will require the segmental resection [6, 7]. Therefore, it is necessary to explore the detailed mechanism of bone invasion of OSCC.

Chemokines and chemokine receptor binding serve a significant role in regulating the progression of cancers in the tumor microenvironment (TME) [8]. C-X-C chemokine receptor type 4 (CXCR4) is chemokine receptor that binds to its ligand with stromal cell-derived factor 1, which serve a significant role in the TME [9]. A previous study has reported that CXCR4 can be used to predict the prognosis of OSCC [10]. Zerumbone can target CXCR4 to regulate the motility and proliferative ability of OSCC [11]. In addition, the CXCR4/C-X-C motif chemokine ligand (CXCL)12 axis has been reported to regulate the epithelial-mesenchymal transition (EMT) process in OSCC [12]. The EMT process serves a key role in the bone invasion process [13]. However, to the best of our knowledge, whether the CXCR4 can regulate bone invasion by OSCC through the EMT process remains unknown.

Therefore, this study aimed to investigate the functional role of CXCR4 in driving bone invasion in OSCC and to elucidate its underlying mechanisms. Using a preclinical murine model of OSCC, we sought to determine: (i) whether pharmacological inhibition of CXCR4 with AMD3100 attenuates mandibular bone destruction; (ii) whether this effect is mediated through the suppression of osteoclast activation; and (iii) whether CXCR4 inhibition impairs the EMT process in cancer cells. Elucidating this pathway may provide a therapeutic rationale for targeting CXCR4 to prevent or treat jawbone destruction in patients with advanced OSCC, thereby potentially improving surgical outcomes and quality of life.

## Materials and methods

### Bioinformatics analysis

The microarray data of OSCC (GSE30784, https://www.ncbi.nlm.nih.gov/geo/query/acc.cgi?acc=GSE30784) were downloaded from the GEO dataset. The differentially expressed genes (DEGs) were analyzed using the R software (V.4.2.2) and the results were presented by the volcano plot. ∣Log fold change (FC)∣>1 and adjusted *P* < 0.05 were considered as the cut-off value. The biological process of CXCR4 was identified using the gene ontology (GO) enrichment analysis by Cytoscape 3.7.2 (https://cytoscape.org/) and results were presented in the bubble plot using R 3.6.2. The adjusted *P* < 0.05 was considered as the cutoff value.

### Cell culture

The human oral cancer cell line HSC-3 was purchased from the Shanghai Yingwan Biotechnology Co., Ltd (cat. no. C1294). The cells were cultured in the DMEM (cat. no. M0101A; Whelab) supplemented with 10% FBS (cat. no. G0411A; Whelab) and 1% antimycotic‑antibiotic (cat. no. G0103; Whelab). The cells were incubated in the 37˚C with 5% CO_2_ and 95% air. The CXCR4 inhibitor AMD3100 (cat. no. HY-10046; MedChemExpress) was purchased and diluted in DMSO.

### Animal model construction

In total, T cell-deficient female BALB/c nude mice(age, 6 weeks) were purchased from the Beijing Deisifu company. Due to the small group size (*n* = 3 per group) and the need to initiate treatment simultaneously, formal randomization was not performed. All mice were allocated to groups from the same batch and cage to minimize baseline variance. Blinding was not applied during histological quantification, and this is explicitly stated here for transparency. The mice were divided into the HSC-3 group and the HSC-3 + AMD3100 group. Each group contained three mice. The HSC-3 cells (1 × 10^6^/100 µl) were injected into subcutaneous of the head of mice [14]. The pre-established inclusion criterion was tumor formation at the injection site within one week post-inoculation; all inoculated mice in this study successfully formed tumors and were included. After 3 weeks, the HSC-3 group were injected with 50 µl PBS whereas HSC-3 + AMD3100 group were injected with 100 µl AMD3100, which were administrated once every other day. In total, 1 week later, the mice were sacrificed by the cervical dislocation without using the any drug. Death of each mice were confirmed by respiratory and cardiac arrest. The maximum tumor size permitted in the present study was 2 cm, which was considered as the human endpoint.

### µCT

The mice were scanned by µCT after fixed treatment (The current of the X-ray tube was set at 200 µA, the voltage was 70 KV, the entire object is scanned, the scanning resolution was 9.033785 μm, the exposure time was 350 msec and the scanning angle is 180˚). Afterwards, the original images were re-constructed using the NRecon software (V1.7.4.2, Bruker). The parameter was Smooth = 5, Beam hardening = 8 and Ring artifacts = 25%. Finally, the data were analyzed using the CT Analyser (V1.20.3.0, Bruker) to calculate the TV (Total Volume), BV (Bone Volume), BV/TV, BS (Bone Surface), Tb.N (Trabecular Number) and Tb.th (Trabecular Thickness).

### H&E staining

The whole head of mice were fixed by 4% PFA and decalcified using 10% EDTA for 4 weeks, with the solution changed every 2–3 days. Subsequently, the samples were made into the wax block by paraffin and cut into 5-µm sections. The sections were firstly dewaxed by xylene and then an alcohol gradient, followed by H&E staining and scanning using by a light microscope (TS2; Nikon Corporation).

### Tartrate-resistant acid phosphatase (TRAP) staining

The sections were firstly dewaxed by the xylene and then an alcohol gradient, before being washed by distilled water for 5 min. Afterwards, the sections were incubated in the 37˚C incubator for 2 h. The sections were further incubated with the TRAP stain in 37˚C for 30 min. Finally, the nuclei were stained by hematoxylin and the sections were scanned using a light microscope. In total, five images were taken per slide for qualification. The TRAP-positive cells on the bone surface were counted using the ImageJ software (National Institutes of Health, V1.51j8).

### Immunohistochemical (IHC) staining

After the antigen retrieval, the sections were firstly blocked by the 5% BSA (Solarbio, A8020) for 30 min in room temperature. The sections were then incubated with primary antibodies against CXCR4 (Bioss, cat. no. bs-20317R), MMP9 (Bioss, cat. no. bs-4593R), Snail (Bioss, cat. no. bs-1371R) and Vimentin (Bioss, cat. no. bs-0756R) in 4˚C overnight. After washing using TBS three times, the sections were incubated with secondary antibodies (cat. no. PV-9000; ZSGB-BIO; OriGene Technologies, Inc.) for 1 h in room temperature. Finally, the sections were visualized by the DAB and scanned using a light microscope (TS2; Nikon Corporation). Each slide was imaged with five fields of view. IHC score = % positive cells x intensity. Score of % positive cells: i) 0–25%, 1; 25–50%, 2; 50–75%, 3; and 75–100%, 4.). Score of intensity: 0: No staining; 1, weak staining; 2, moderate staining; and 3, strong staining).

### Statistical analysis

The statistical analysis in the present study was conducted using the Graphpad Prism 9.0 (Dotmatics) and the data were presented as the mean ± standard deviation. The sample size (*n* = 3 per group) was determined by practical constraints for this preliminary, hypothesis-generating study. This design is suited for detecting large effect sizes (Cohen’s d > 2.5), which are of primary interest in early mechanistic investigations. The comparation between two groups was conducted using the Unpaired Student’s t-test.Where applicable, 95% confidence intervals (CIs) are reported to provide estimates of effect precision. For the analysis of correlated endpoints (e.g., multiple micro-CT parameters), results are presented descriptively to comprehensively characterize the bone phenotype. For the distinct yet biologically related EMT markers, p-values are reported without formal multiplicity adjustment to avoid an undue increase in type II error, with the interpretation focused on the consistency and magnitude of the observed effects. *P* < 0.05 was considered to indicate a statistically significant difference.

## Results

### CXCR4 is upregulated in OSCC and linked to invasion-associated processes

**Fig. 1:**
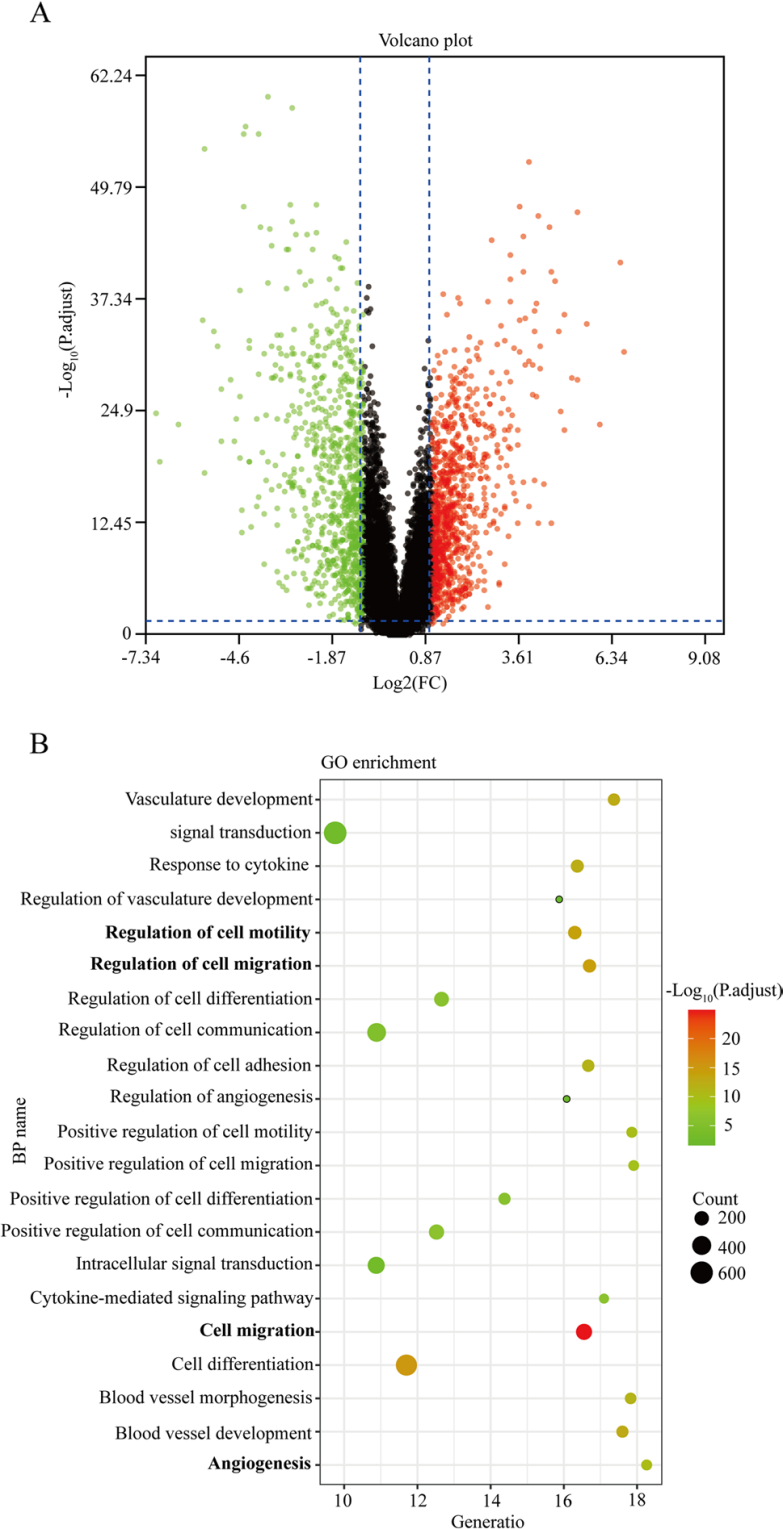
Prediction of CXCR4-associated biological processes in the OSCC. **A** The differentially expressing genes in the OSCC were identified by the R software and presented as volcano plots. **B** The CXCR4-associated biological processes were presented by the bubble plot. CXCR4, C-X-C chemokine receptor type 4; OSCC, oral squamous cell carcinoma

The microarray data of OSCC, GSE30784, was downloaded from the GEO dataset and analyzed by the R software, which indicated that CXCR4 expression was upregulated in the OSCC tissues compared with that in the normal oral mucosa tissues (Fig. 1A). The biological processes of CXCR4 that were enriched was analyzed using the GO enrichment analysis, which revealed that CXCR4 was mainly enriched in the cell motility, cell migration and angiogenesis processes (Fig. 1B). These data suggest that the upregulated CXCR4 expression is associated with bone invasion by OSCC.

### Inhibition of CXCR4 can inhibit the bone invasion of OSCC in vivo

**Fig. 2:**
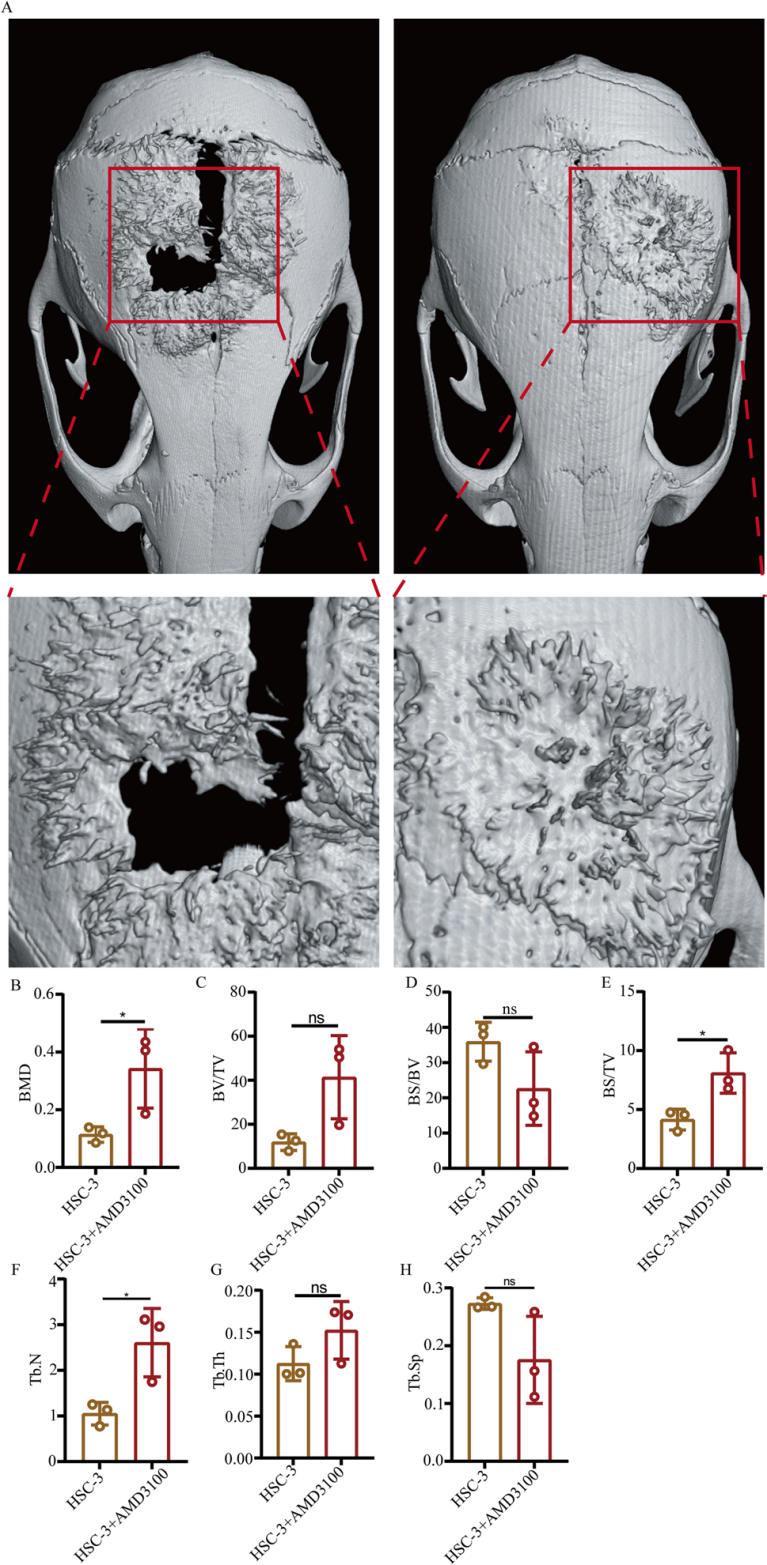
Examination of the effect of the CXCR4 inhibitor on the bone resorption degree by HSC-3 cells in vivo. **A** The effect of CXCR4 inhibitor on bone resorption by HSC-3 in vivo was examined using µCT. **B**-**H** Semi-quantification of bone resorption degree according to different indicators. Data are shown as mean ± standard deviation and analyzed using the unpaired student’s t-test. ^*^P < 0.05. CXCR4, C-X-C chemokine receptor type 4

The effect of the CXCR4 inhibitor on the bone invasion of OSCC was next examined using µCT (Fig. 2A). The Bone Mineral Density (BMD) in the HSC-3 + AMD3100 group was found to be higher compared with that in the HSC-3 group (Fig. 2B). The ratio of BV/TV in the HSC-3 + AMD3100 group was also found to be higher compared with that in the HSC-3-only group (Fig. 2C). By contrast, the ratio of BS/BV in the HSC-3 + AMD3100 group was observed to be lower compared with that in the HSC-3-only group (Fig. 2D). In addition, the ratio of BS/TV in the HSC-3 + AMD3100 group was higher compared with that in the HSC-3 only group (Fig. 2E). Both the Tb.N and Tb.Th in the HSC-3 + AMD3100 group were higher compared with that in the HSC-3-only group (Fig. 2F and G). The Tb.sp in the HSC-3 + AMD3100 group was lower compared with that in the HSC-3-only group (Fig. 2H). All these data suggest that the inhibition of CXCR4 can inhibit the bone invasion of OSCC in vivo.

### Inhibition of CXCR4 inhibits bone invasion by OSCC by suppressing the activation of osteoclasts

**Fig. 3:**
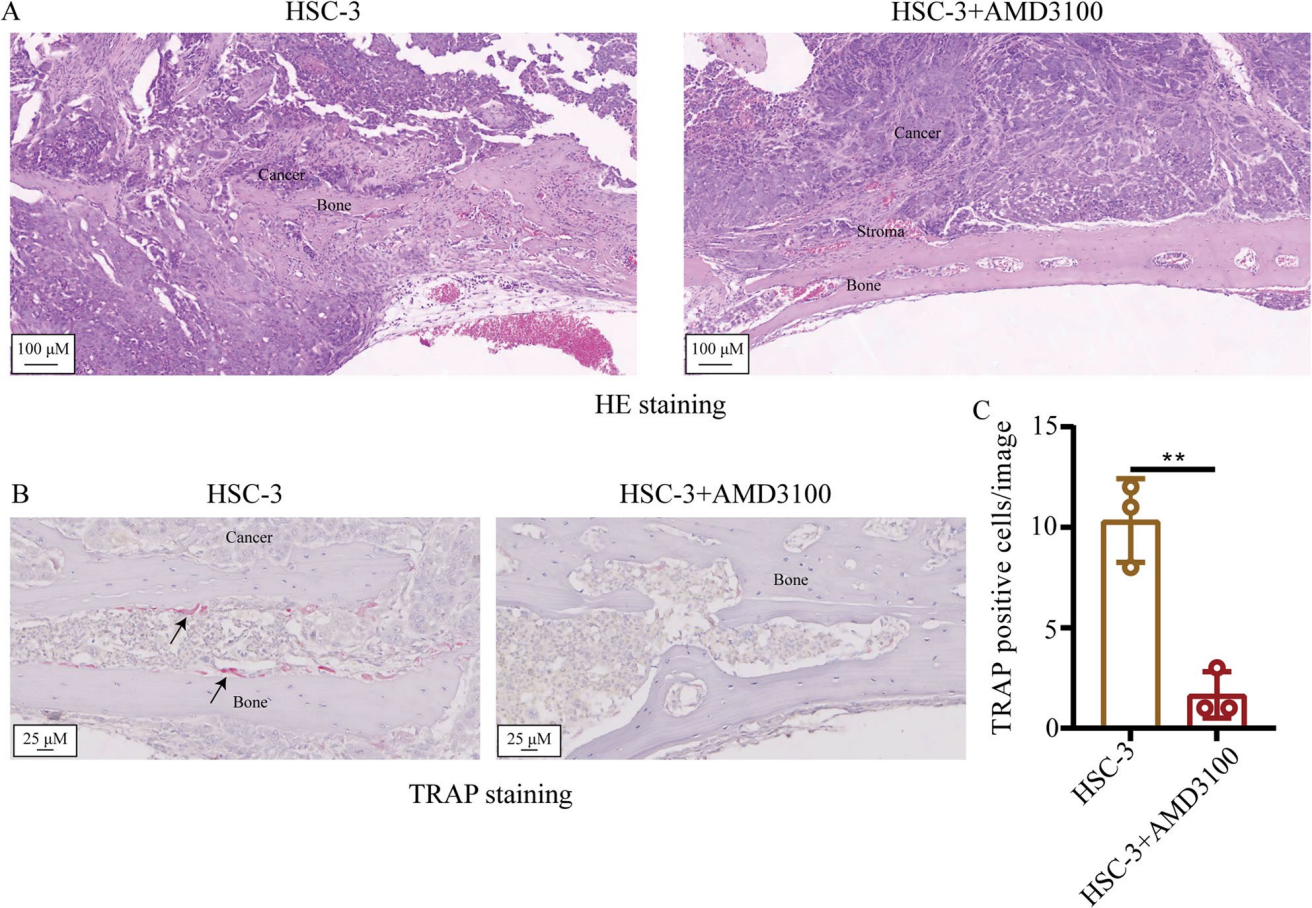
Examination of the effect of the CXCR4 inhibitor on the activation of osteoclasts in the microenvironment of HSC-3 in vivo. **A** H&E staining was used to present the histological findings in the different groups. **B** TRAP staining was used to present the TRAP-positive cells in the different groups. **C** Quantification of TRAP-positive cells in different groups. Black arrow indicated the TRAP-positive cells. Data are shown as mean ± standard deviation and analyzed using the unpaired student’s t-test. ^**^P < 0.01. CXCR4, C-X-C chemokine receptor type 4; TRAP, tartrate-resistant acid phosphatase

The effect of the CXCR4 inhibitor on the bone invasion degree by HSC-3 in vivo was next tested by H&E staining. The results indicated that the bone resorption degree in the HSC-3 group was more serious compared with that in the HSC-3 + AMD3100 group (Fig. 3A). The effect of CXCR4 inhibitor on the activation of osteoclasts was examined by observing the proportion of TRAP-positive cells (Fig. 3B). The number of TRAP-positive cells in the HSC-3 + AMD3100 group was reported to be lower compared with that in the only HSC-3 group (Fig. 3C). These data suggest that use of the CXCR4 inhibitor inhibited bone invasion by OSCC by inhibiting the activation of osteoclasts.

### Inhibition of CXCR4 suppresses the expression of invasion and EMT markers in OSCC

**Fig. 4:**
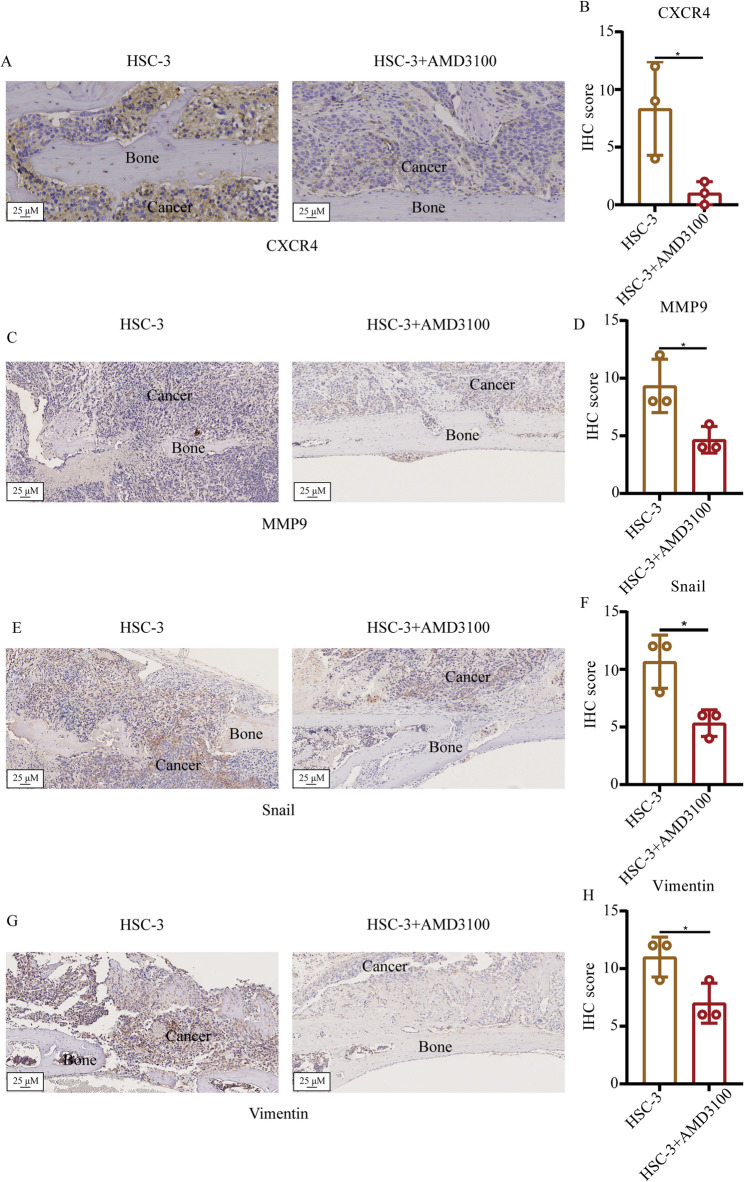
Examination of the effect of the CXCR4 inhibitor on the invasion and migration of HSC-3 cells in vivo. **A**, **C**, **E** and **G** Immunohistochemical staining was used to test the expression of CXCR4 (**A**), MMP9 (**C**), Snail (**E**) and vimentin (**G**) in the HSC-3 in vivo. **B**, **D**, **F** and **H** Semi-quantification of CXCR4 (**B**), MMP9 (**D**), Snail (**F**) and vimentin (**H**) expression in different groups. Data are shown as mean ± standard deviation and analyzed using the unpaired student’s t-test. ^*^P < 0.05. CXCR4, C-X-C chemokine receptor type 4

Given that bone invasion by OSCC is mainly mediated by the invasion and migratory ability of cancer cells [5], the invasion biomarker MMP9 and EMT biomarkers Snail and vimentin were next examined by IHC staining (Fig. 4C, E and G), which indiated that the IHC score of MMP9, Snail and Vimentin in HSC-3+AMD3100 group was lower than that in the only HSC-3 group (Fig. 4D, F and H).The IHC score for CXCR4 itself was confirmed to be significantly lower in the HSC-3 + AMD3100 group compared to the HSC-3-only group (Fig. 4A and B, *p* < 0.05), verifying the efficacy of the inhibitor.

Critically, a concomitant and marked downregulation was observed for all three pro-invasive markers following CXCR4 inhibition. The IHC scores for MMP9, Snail, and Vimentin were reduced by 30–50% in the treatment group compared to controls (all unadjusted *p* < 0.05). Given the exploratory nature of this coordinated analysis and the consistent direction of the profound effects across all related markers, p-values are reported without formal correction for multiple comparisons, with the emphasis placed on the large and biologically coherent effect sizes observed.

These findings, revealing a concerted suppression of the molecular machinery that drives cell invasion and motility, provide a plausible mechanism by which CXCR4 inhibition could directly impair the local invasive capacity of OSCC cells, an effect that may operate alongside any potential reduction in overall tumor growth.

## Discussion

Oral squamous cell carcinoma is a common malignant tumor of the head and neck region, with > 300,000 new cases worldwide each year and is characterized by a high degree of aggressiveness and metastasis [15]. A major clinical challenge arises when OSCC, originating in sites adjacent to the jawbone, invades the mandible. This bone invasion frequently necessitates extensive surgery, severely impacting patients’ quality of life and signifying a poor prognosis [16, 17]. In this study, we demonstrate that pharmacological inhibition of CXCR4 with AMD3100 significantly suppresses mandibular bone invasion in a murine model of OSCC. Our key findings are that CXCR4 blockade attenuates osteoclast activation at the tumor-bone interface and concurrently inhibits the epithelial-mesenchymal transition (EMT) program within cancer cells.

Bone invasion by OSCC is not directly mediated by tumor cells, but by osteoclasts [18]. Osteoclast formation and activation are regulated by receptor activator of NF-κB ligand (RANK) ligand (RANKL), RANK and osteoprotegerin (OPG). OPG functions as a decoy receptor for RANKL and can inhibit its binding to RANK by binding to RANKL, thereby inhibiting bone resorption. The balance between RANKL and OPN expression serves a key role in osteoclast differentiation and function, where dysregulation of the ratio between the two has been found in a variety of bone metabolism diseases such as postmenopausal osteoporosis [19–21]. OSCC can also secrete a variety of cytokines such as the PTHrP, which are directly or indirectly involved in the regulation of the RANKL/RANK signaling pathway, regulating the formation and activation of osteoclasts, hence invasion of the jawbone. In the TME of OSCC bone invasion, tumor cells, osteoclasts and fibroblasts can mutually interact with and promote each other [22]. In recent years, the role of chemokines has received attention. Chemokines are short, heparin-like binding peptides that belong to a superfamily of cytokines with chemotactic effects on leukocytes and can promote cell migration. In previous study, it has been shown to be strongly associated with tumor proliferation and metastasis [23]. CXCR4 belongs to the CXCR family of chemokine receptors and is a G-protein-coupled receptor. It is typically either not expressed or poorly expressed in normal tissues, but is widely expressed in > 20 human cancers, including colorectal, breast, ovarian, melanoma and prostate cancers [24]. CXCL12 is a classical ligand for CXCR4, where the specific binding between the two to form the CXCL12/CXCR4 axis is the molecular basis for its biological function. During tumor development, tumor cells with high CXCR4 expression can spread or metastasize to tissues or organs with high CXCL12 expression through chemotaxis [25]. Previous studies have reported that there is a crosstalk between lipid metabolism and bone homeostasis, where adipocytes can promote osteoclast differentiation and function through the CXCL12/CXCR4 signaling pathway [26, 27].

Our findings on osteoclast activation can be viewed in the context of recent research. Studies have confirmed that in OSCC-associated bone destruction, osteoclast activation is linked not only to carcinoma cells but also closely to stromal cells within the tumor microenvironment, with stromal cells in more invasive cancers exerting a stronger pro-osteoclastogenic effect. Furthermore, genomic analyses have identified several potential mediators of bone invasion, including the CXCR4 ligand, CXCL12 [28]. In our research, CXCR4 serves as the classical receptor for CXCL12. Our data provide direct in vivo evidence that targeting CXCR4 itself is sufficient to suppress osteoclast activation and subsequent bone resorption, thereby extending previous work focused on the general biology of osteoclasts in the tumor microenvironment.

The EMT process has an important role in OSCC bone invasion, where previous study has demonstrated that the TGF-β signaling pathway can mediate the EMT process and is involved in jawbone invasion by oral cancer through the RANKL-induced activation of osteoclasts [29]. In addition, the ononin inhibited bone metastasis by breast cancer by inhibiting the EMT process [30]. Therefore, the EMT process can serve a significant role in bone invasion and metastasis by multiple cancers. CXCL12/CXCR4 is overexpressed in OSCC and is closely associated with its development and migration. Previous studies have demonstrated that CXCL12/CXCR4 can induce EMT in OSCC [31, 32]. AMD3100 acts as a CXCR4 antagonist and can competitively inhibit the binding of CXCL12 to CXCR4. In the present study, bioinformatics analysis revealed that CXCR4 is highly expressed in OSCC, which was in turn associated with bone invasion. In our model of OSCC bone invasion, treatment with AMD3100 significantly suppressed the degree of bone invasion, as assessed by imaging and histology. we found that the degree of bone invasion by OSCC was significantly suppressed. Inhibition of CXCR4 suppresses osteoclast activation, thereby inhibiting bone invasion by OSCC. Finally, and most critically, we found that the protein expression of MMP9, Snail, and Vimentin was suppressed after CXCR4 inhibition. The downregulation of these specific molecules, which are established executors of matrix degradation and cellular motility, provides a direct mechanistic link whereby CXCR4 inhibition impairs the invasive capacity of OSCC cells, an effect that can be dissociated from a general reduction in tumor growth.

Beyond the mechanistic insights, our findings hold significant clinical promise. AMD3100 (Plerixafor) is an FDA-approved drug for stem cell mobilization, which could facilitate its repurposing for oncology applications. For patients with OSCC at high risk of bone invasion, a CXCR4-targeting strategy could be explored as a neo-adjuvant or adjunctive therapy to shrink tumors and protect the jawbone integrity, potentially enabling less disfiguring surgeries and improving post-treatment quality of life. Future clinical trials are warranted to evaluate the safety and efficacy of CXCR4 inhibitors in combination with standard-of-care treatments for OSCC.

In summary, data from the present study suggests that CXCR4 inhibition can suppress bone invasion by OSCC through a dual mechanism. Although the potential impact on overall tumor size remains a consideration, our results indicate that CXCR4 blockade can directly reduce the degree of jawbone destruction by inhibiting the activity of osteoclasts, and can also reduce the expression of EMT-related proteins, thereby acting on the EMT process to reduce the metastatic and invasive potential of OSCC. Therefore, these experimental results imply that CXCR4 is a potential therapeutic target for the treatment of OSCC bone invasion and osteolysis.

## Data Availability

The data generated in the present study may be requested from the corresponding author.
